# The Terrible Triad of Checkpoint Inhibition: A Case Report of Myasthenia Gravis, Myocarditis, and Myositis Induced by Cemiplimab in a Patient with Metastatic Cutaneous Squamous Cell Carcinoma

**DOI:** 10.1155/2020/5126717

**Published:** 2020-07-04

**Authors:** Nikeshan Jeyakumar, Mikel Etchegaray, Jason Henry, Laura Lelenwa, Bihong Zhao, Ana Segura, L. Maximilian Buja

**Affiliations:** ^1^Division of Internal Medicine, The University of Texas M.D. Anderson Cancer Center, 1400 Pressler Drive, Unit 1463, Houston, Texas 77030, USA; ^2^Division of Cancer Medicine, The University of Texas M.D. Anderson Cancer Center, 1515 Holcombe Blvd, Houston, Texas 77030, USA; ^3^Department of Pathology and Laboratory Medicine, The University of Texas Health Science Center at Houston, McGovern Medical School, 6431 Fannin Street, P.O. Box 20708, Houston, Texas 77225, USA; ^4^Department of Cardiovascular Pathology, Texas Heart Institute, 6770 Bertner Avenue, Houston, Texas 77030, USA

## Abstract

**Background:**

We report a case of a patient with squamous cell carcinoma (SCC) who developed myasthenia gravis (MG), myositis, and myocarditis after receiving cemiplimab, an anti-PD-1 immune checkpoint inhibitor (ICI). *Case Presentation.* An 86-year-old man with metastatic periocular SCC presented with decreased vision in the left eye, severe fatigue, and lower back and bilateral hip pain 3 weeks after receiving cemiplimab. Within hours, he developed dysphonia, pharyngeal secretions, and dysphagia, necessitating intubation. Endomyocardial biopsy revealed active lymphocyte-mediated necrosis consistent with ICI-induced myocarditis. Anti-striated muscle and anti-acetylcholine receptor antibodies were elevated, consistent with myositis and myasthenia gravis. Despite plasma exchange therapy, steroids, and intravenous immunoglobulin, he died from cardiac arrest.

**Conclusions:**

The presence of myasthenia gravis, myocarditis, or myositis should prompt evaluation for all three toxicities as they may represent an overlap syndrome. The severity of these immunotoxicities highlights the need for clinicians to suspect multiple simultaneous adverse effects of ICIs.

## 1. Introduction

Cemiplimab is a programmed cell death protein 1 (PD-1) immune checkpoint inhibitor (ICI) approved in September 2018 for the treatment of locally advanced or metastatic cutaneous squamous cell carcinoma (SCC) in patients that did not qualify for curative surgery or radiation [1]. Phase 2 studies of patients with metastatic disease demonstrated a response rate of 47% (28/59), with 16 (57%) of these patients having duration of response at 6 months [2]. The most common immune-related adverse events (irAEs) were diarrhea, fatigue, constipation, and rash [2]. Neuromuscular irAEs occur in <1% of patients treated with ICIs overall [3], but due to the nonspecific nature of their symptoms, it is likely that many cases go unreported [4]. Myasthenia gravis (MG) is the most commonly reported neuromuscular irAE associated with PD-1 inhibitors, with incidence ranging from 0.12 to 0.2% [5]. PD-1 inhibitor-related MG tends to be more severe, with 40–50% of patients requiring ventilatory support (7 times higher than in typical MG) [5]. There also appears to be a distinct correlation between PD-1 inhibition and MG, as MG is rarely seen in association with anticytotoxic T-lymphocyte-associated protein 4 (CTLA-4) therapy. In a review of over 10,000 patients treated with nivolumab or ipilimumab in Japan, 12 cases of MG occurred in patients who received nivolumab, while none occurred with ipilimumab [6]. Ten of these 12 were seropositive for acetylcholine receptor (AChR) antibodies [6]. ICI-related MG is also strongly associated with elevated creatine kinase (CK) levels, indicating the presence of simultaneous muscle or cardiac dysfunction [5]. The rare and generally fatal triad of ICI-related MG, myocarditis, and myositis has been described with nivolumab/ipilimumab dual therapy [3] and pembrolizumab [7] and nivolumab monotherapy [6, 8] but has not previously been reported with cemiplimab.

## 2. Case Presentation

An 86-year-old man with periocular SCC involving the left lower and upper eyelids status after Mohs surgery, reconstruction, and adjuvant radiation therapy was referred to the MD Anderson Cancer Center due to local recurrence of SCC to the left lateral canthus and orbit. He had a prior history of numerous cutaneous carcinomas (basal cell, spindle cell, and squamous cell) on the head and face, removed surgically. His comorbidities included a 4-vessel coronary artery bypass graft in 2016, sick sinus syndrome status after pacemaker placement, hypertension, hyperlipidemia, and chronic kidney disease (CKD) with creatinine clearance (CrCl) 22 mL/min. Outpatient ultrasound-guided biopsy of left parotid and submandibular nodules revealed metastatic SCC in both areas. After discussion with head and neck medical oncology, the patient elected to start cemiplimab and received one 350 mg dose.

Three weeks later, he reported to the emergency room with 5 days of decreased vision in the left eye and a 48-hour history of severe fatigue accompanied by lower back and bilateral hip pain. He had difficulty arising from his chair but denied double vision, difficulty swallowing or walking, muscle aches or tenderness, shortness of breath, chest pain, fevers, chills, nausea, vomiting, diarrhea, or bowel/bladder dysfunction. Vital signs were normal. Physical exam was notable for right-sided ptosis and a large, firm mass lateral to the left orbit, causing unilateral proptosis and extending through the ipsilateral parotid and submandibular region. Distance vision in the left eye was slightly diminished, but pupillary responses and extraocular reflexes were intact bilaterally.

Cranial nerve testing was unremarkable. Cardiac, lung, and abdominal exams were within normal limits. He had proximal muscle weakness primarily in the lower extremities but did not have tenderness to palpation of major muscle groups or fatiguability. He demonstrated a normal gait.

Computed tomography imaging of the hip and lumbar spine did not show any metastatic disease or cord compression (magnetic resonance imaging could not be done due to the patient's pacemaker). Notable initial laboratory abnormalities are listed in Table 1.

An electrocardiogram was suggestive of inferior wall ischemia and showed a new right bundle-branch block. Transthoracic echocardiogram showed a normal ejection fraction without pericardial effusion.

**Figure 1:**
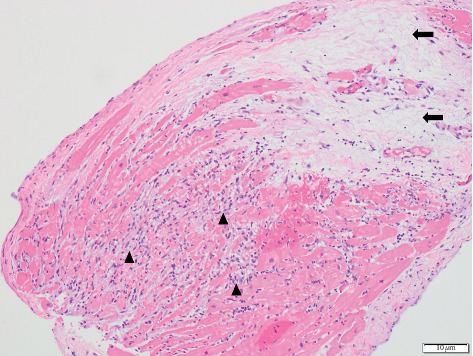
Endomyocardial biopsy showing evidence of acute necrotizing myocarditis with inflammatory cellular infiltrate and cardiomyocyte necrosis (arrowheads) and focal replacement fibrosis (arrows) indicative of previous damage and healing.

Several hours after initial presentation, the patient developed dysphonia, increasing pharyngeal secretions and difficulty swallowing, and new shortness of breath with worsening fatigue. He was urgently transferred to the intensive care unit, given 1 g intravenous methylprednisolone, and was electively intubated. Left and right heart catheterization with endomyocardial biopsy was performed and revealed an active necrotizing lymphocytic myocarditis (Figure 1). His bypass grafts were patent, and he had normal ventricular pressures. Results of autoimmune antibody testing are shown in Table 1. Muscle-specific kinase antibodies were negative.

He received plasma exchange therapy for 5 days, was continued on high-dose methylprednisolone, and received one dose of intravenous immunoglobulin. However, his troponin-T levels continued rising over 3000 ng/L, his troponin-I levels peaked at 6.9 ng/L, and he began developing worsening kidney function necessitating initiation of continuous renal replacement therapy. Despite these measures, he suffered pulseless electrical activity arrest from hyperkalemia and severe metabolic acidosis and could not be resuscitated. Based on his clinical signs and symptoms, elevations in anti-AChR antibodies, anti-striated muscle antibodies, CK, and urine myoglobin, and with characteristic findings on endomyocardial biopsy, he was diagnosed with cemiplimab-related MG, myositis, and myocarditis.

## 3. Discussion

This case was illustrative of the varied and often vague presentations of irAEs. This patient did not have chest pain, dyspnea, or any other symptoms consistent with cardiac damage despite his significant cardiac history and highly elevated troponins on presentation. His echocardiogram was also normal, which necessitated cardiac catheterization and endomyocardial biopsy for diagnosis. Another remarkable finding was the speed of his clinical deterioration; over a few hours, he went from being minimally symptomatic to needing intubation for deteriorating pulmonary mechanics and bulbar dysfunction. His rapid decline required swift coordination of various hospital services for him to receive emergent plasma exchange and immunosuppression.

This patient's renal function was below the limit tested in cemiplimab clinical trials (CrCl > 25 mL/min) [1]. However, ICIs are typically broken down and cleared by phagocytic cells, so renal function is not expected to affect drug activity or metabolism [9]. A study of 27 patients with preexisting renal, hepatic, and/or cardiac dysfunction showed that treatment with PD-1 inhibitors does not typically result in excessive irAEs or increased organ damage [10]. Nonetheless, the combination of this patient's advanced age, frailty, and multiple comorbidities likely predisposed him to a worse outcome.

To our knowledge, this is the first reported case of simultaneous MG, myocarditis, and myositis occurring secondary to cemiplimab. A recent review of ICI-related neuromuscular toxicities reports that MG is the most common neuromuscular irAE (26.8%, *n* = 22), followed by myositis (25.6%, *n* = 21) and Guillain–Barre syndrome (18.3%, *n* = 15) [4]. Myositis (16.2%) and myocarditis (8.8%) were the most common immunotoxicities accompanying cases of ICI-induced MG [11]. The presence of all 3 toxicities was associated with a significantly higher risk of death (5/8 patients; 62.5%) than MG alone (29/179; 16.2%), MG + myositis (6/29; 20.7%), or myocarditis alone (4/12; 33%) [11]. MG also tended to occur earlier (median 29 days after immunotherapy) than other neurotoxicities [11]. A report of 101 cases of ICI-related myocarditis showed that myositis (25%) and MG (10%) were the two most common concurrent irAEs [12]. Complicating this picture is that ICI-related myositis often presents with oculomotor weakness [13], which can confound the diagnosis of MG. Our patient's laboratory and pathologic findings are consistent with prior reports of “PD-1 myopathy,” a discrete inflammatory myopathy observed in patients who received nivolumab or pembrolizumab and subsequently developed muscle weakness. These patients had an average CK of 5247 IU/L and multifocal myonecrosis with endomysial inflammation on histologic examination [14]. Thirteen of 19 patients (68%) also had elevated anti-striated muscle antibodies [14]. Several other groups have also noted increases in anti-striated muscle antibodies associated with myopathy after PD-1 ICI therapy [15–17]. Our case adds to this growing body of evidence suggesting that anti-striated muscle antibodies are sensitive markers of PD-1 myopathy and should be checked in patients with symptoms of myositis in the context of recent PD-1 ICI exposure.

Neuromuscular irAEs such as MG may be part of an overlap syndrome with myocarditis and myositis [4]. MG has also been seen more frequently with anti-PD-1 and antiprogrammed cell death ligand 1 (PD-L1) ICIs compared to anti-CTLA-4 ICIs [11]. Mechanistically, this may be partially explained by the differing distribution of PD-1 and CTLA-4 in immune cells. PD-1 is expressed on B cells, T cells, and myeloid cells, whereas CTLA-4 is typically localized to T cells [18]. The increased incidence of MG in anti-PD-1 therapy compared to anti-CTLA-4 therapy thus may in part be due to PD-1 blockade on B cells, resulting in proliferation of autoreactive B cells that create antibodies to self-antigens on the AChR and neuromuscular junction. Evidence for this mechanism comes from an *in vitro* study showing that B cells widely express both PD-1 and PD-L1, and that PD-1/PD-L1 binding decreases B-cell activation, proliferation, and interleukin-6 synthesis [19]. PD-1 blockade then restores and enhances B-cell proliferation and cytokine production [19]. In contrast, CTLA-4 blockade results in broad activation and proliferation of T cells and simultaneous reduction of regulatory T-cell-mediated immunosuppression [18], without direct impact on B cells. In general, irAEs from both anti-PD-1/PD-L1 and anti-CTLA-4 therapies are largely similar and most commonly impact the gastrointestinal, dermatologic, and endocrine systems [20], indicating that T cells are the primary driver of both efficacy and toxicity. However, rituximab has been shown to be useful in resolving immune thrombocytopenic purpura after ICI therapy [21], suggesting that abrogating B-cell activity may have some benefit in treating similar antibody-mediated conditions such as ICI-related MG. Further investigation into the dynamics between PD-1 inhibitors and B cells is warranted.

The presence of any one of MG, myocarditis, or myositis should prompt immediate evaluation for all three toxicities. Both troponin-T and troponin-I should be ordered to better differentiate cardiac from skeletal muscle damage. Anti-striated muscle, anti-AChR, and muscle-specific kinase antibodies may be helpful in differentiating specific toxicities. Endomyocardial biopsy should also be performed for diagnosis as electro- and echocardiogram may not reveal dysfunction. The rapid progression of these adverse effects requires quick identification and initiation of immunosuppression and plasma exchange. The unusual presentation and severity of this triad of immunotoxicities also highlight the need for physicians to be alert for multiple simultaneous irAEs after ICI therapy.

## Figures and Tables

**Table 1:** Notable abnormal labs on admission and results of autoimmune testing.

Labs on admission	Recorded values	Normal values
Creatine kinase (CK)	6,407 IU/L	22–198 IU/L
High-sensitivity troponin-T	1,557 ng/L	< ng/L
Potassium	5.8 mEq/L	3.5–5.0 mEq/L
Aspartate aminotransferase	532 IU/L	<40 IU/L
Alanine aminotransferase	214 IU/L	<40 IU/L
Urine myoglobin	20 ng/mL	<5 ng/mL
Creatinine	2.14 mg/dL	1.5 mg/dL (for this patient)

*Autoimmune antibody testing*		
Anti-striated muscle antibody titers	1:15,360	<1:120
Acetylcholine receptor antibody levels	9.34 nmol/L	<0.02 nmol/L
Anti-Ro antibody levels	51 U/mL	<20 U/mL

## Data Availability

Primary data about the patient were obtained from the electronic medical record of the University of Texas MD Anderson Cancer Center. Cited manuscripts were found on PubMed.

## Abbreviations

AChR: Acetylcholine receptor
CK: Creatine kinase
CKD: Chronic kidney disease
CrCl: Creatinine clearance
CTLA-4: Cytotoxic T-lymphocyte-associated protein 4
ICI: Immune checkpoint inhibitor
irAE: Immune-related adverse event
MG: Myasthenia gravis
PD-1/PD-L1: Programmed cell death protein 1/ligand 1
SCC: Squamous cell carcinoma.
